# Pre-clinical 2D and 3D toxicity response to a panel of nanomaterials; comparative assessment of NBM-induced liver toxicity

**DOI:** 10.1007/s13346-022-01170-1

**Published:** 2022-06-28

**Authors:** Melissa Anne Tutty, Gabriele Vella, Adriele Prina-Mello

**Affiliations:** 1 Nanomedicine and Molecular Imaging Group, Trinity Translational Medicine Institute (TTMI), School of Medicine, Trinity College Dublin, Dublin 8, Ireland; 2 Laboratory for Biological Characterisation of Advanced Materials (LBCAM), TTMI, School of Medicine, Trinity College Dublin, Dublin 8, Ireland; 3 Trinity St James’s Cancer Institute, Trinity College Dublin, St James’s Hospital, Dublin 8, Ireland

**Keywords:** Nanobiomaterials, NBMs, 3D culture, Spheroids, Cytotoxicity, Viability, HepG2, Liver spheroid, 3Rs, DILI, Hepatotoxicity

## Abstract

Nanobiomaterials, or NBMs, have been used in medicine and bioimaging for decades, with wide-reaching applications ranging from their uses as carriers of genes and drugs, to acting as sensors and probes. When developing nanomedicine products, it is vitally important to evaluate their safety, ensuring that both biocompatibility and efficacy are achieved so their applications in these areas can be safe and effective. When discussing the safety of nanomedicine in general terms, it is foolish to make generalised statements due to the vast array of different manufactured nanomaterials, formulated from a multitude of different materials, in many shapes and sizes; therefore, NBM pre-clinical screening can be a significant challenge. Outside of their distribution in the various tissues, organs and cells in the body, a key area of interest is the impact of NBMs on the liver. A considerable issue for researchers today is accurately predicting human-specific liver toxicity prior to clinical trials, with hepatotoxicity not only the most cited reasons for withdrawal of approved drugs, but also a primary cause of attrition in pre-launched drug candidates. To date, no simple solution to adequately predict these adverse effects exists prior to entering human experimentation. The limitations of the current pre-clinical toolkit are believed to be one of the main reasons for this, with questions being raised on the relevance of animal models in pre-clinical assessment, and over the ability of conventional, simplified in vitro cell–based assays to adequately assess new drug candidates or NBMs. Common 2D cell cultures are unable to adequately represent the functions of 3D tissues and their complex cell–cell and cell–matrix interactions, as well as differences found in diffusion and transport conditions. Therefore, testing NBM toxicity in conventional 2D models may not be an accurate reflection of the actual toxicity these materials impart on the body. One such method of overcoming these issues is the use of 3D cultures, such as cell spheroids, to more accurately assess NBM-tissue interaction. In this study, we introduce a 3D hepatocellular carcinoma model cultured from HepG2 cells to assess both the cytotoxicity and viability observed following treatment with a variety of NBMs, namely a nanostructured lipid carrier (in the specific technical name = LipImage^™^ 815), a gold nanoparticle (AuNP) and a panel of polymeric (in the specific technical name = PACA) NBMs. This model is also in compliance with the 3Rs policy of reduction, refinement and replacement in animal experimentation [1], and meets the critical need for more advanced in vitro models for pre-clinical nanotoxicity assessment.

**Graphical abstract:**

Pipeline for the pre-clinical assessment of NBMs in liver spheroid model

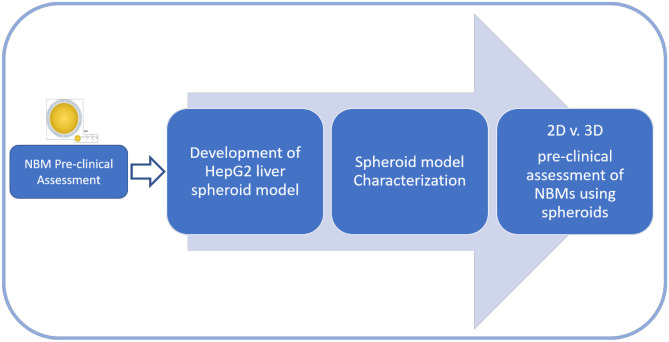

**Supplementary Information:**

The online version contains supplementary material available at 10.1007/s13346-022-01170-1.

## Introduction

Nanoparticles (NPs) are used today for a wide variety of applications ranging from the fields of textiles and engineering to cosmetics, food and specifically medicine, where they are used in diagnostics, biosensing, drug delivery and therapeutics [2, 3]. Due to their far-reaching applications, increasing use and intention that they will be delivered to the human body, NPs used for biomedical applications, or NBMs, must have their potential fates and adverse effects considered and rigorously tested to achieve overall safety and acceptance. NBMs are primarily administered via inhalation, ingestion, dermal administration and IV injection, whereby they are distributed all over the body, before accumulation in key secondary organs such as the spleen and liver [2, 4, 5].

With regard to NBM safety assessment, for decades, the gold standard has been a barrage of in vitro assays based upon 2D cell cultures; however, there is increasing information to suggest that these models do not appropriately mimic human responses and discrepancies can be seen between in vitro and in vivo pre-clinical assessments. A 3D environment is essential for cells to grow and metabolise correctly [6], with the function and phenotype of individual cells highly dependent on the complex interactions which occur between the 3D structure of the extracellular matrix (ECM) and its neighbouring cells [7]. When cultured in two dimensions however, these complex cell–cell and cell–matrix interactions are impeded, which has a knock-on effect in recapitulating accurate and predictive in vivo cellular responses [8]. Therefore, whilst 2D cell cultures are cheap to use and easy to maintain and work with, and whilst they have provided invaluable information on the cellular responses imparted by drugs and NBMs for decades, tests which are based upon 2D in vitro cell culture models do not accurately predict toxicity of NBMs due to their lack of key physiological processes, including transport of these materials through cells [7, 9]. This is further emphasised in many studies, including work on the toxicity screening of quantum dots [10], carbon nanotubes [11] and magnetic NPs [12] whereby significant cytotoxicity was induced in 2D in vitro cell cultures; however, this effect was not mimicked in animal models where no adverse effects were observed [13–15].

The large discrepancies between 2D cell culture and animal models are becoming increasingly apparent, and in recent years, more emphasis has been placed on advanced in vitro methodologies for assessing interactions and possible effects of NBMs [16], not only due to the fact that traditional in vitro cell models lack the phenotypic details of the in vivo environment, but also because physiological function and crosstalk between cells also are highly reduced in traditional models. 3D models, such as cellular spheroids, have been described as a potential methodology to overcome these issues, bridging the gap between traditional 2D models and in vivo animal work, and acting as better predictors of NBM toxicity [17]. Various new mono- and co-culture 3D models have been developed, combining different relevant cell types into one organotypic model which can be used to determine not only NBM toxicity, but also their interactions with various cell types in a 3D environment [16]. Whilst the majority of 3D models are still not standardised or validated methods for assessing the cellular interactions and toxicities associated with NBMs, in recent years, many studies have used them in pre-clinical investigation, for applications in both the optimisation of NBM physicochemical properties and screening of their therapeutic effects [18].

Due to the central role the liver plays in the metabolism, clearance and biotransformation of drugs and NBMs, another consideration for researchers today has been predicting human-specific liver toxicity [19]. As a means of reducing both time and cost, over many decades, a vast array of in vitro methodologies have been developed to test the liver toxicity of NBMs. These models have led to many important insights into toxicity, safety and efficacy of these materials, and they are essential tools in both the discovery and pre-clinical stages of drug/NBM development [20, 21]. For decades, in vitro 2D cultures of the liver have been the gold standard for determining acute hepatotoxicity of NBMs. Currently, 2D in vitro assays are based on either primary cell cultures derived directly from harvested liver tissue or immortalised hepatic cell lines, i.e. primary cells which have been genetically transformed to generate rapidly proliferating, easily cultured cells with artificial phenotypes that can be grown for prolonged periods. Being useful early predictors of toxicity, these immortalised cell lines have reduced metabolic competencies in comparison to their primary hepatocyte counterparts. Therefore, in recent years, there has been a small push towards using 3D liver spheroid cultures for assessing the impact of NBMs. To date however, the effect of each of the chosen NBMs presented in this study on a 3D hepatocellular carcinoma model has not yet been discussed in literature. Here, for the first time, a dye-loaded nanostructured lipid carrier (LipImage^™^ 815), a 20 nm PEGylated gold NBM (AuNP) and three poly(alkyl cyanoacrylate) (PACA) NBM formulations (i.e. unloaded, dye-loaded and drug (CBZ) loaded) were characterised, before their ability to impact HepG2 cell and spheroid viability and their ability to induce cytotoxicity were assessed in both models.

## Materials and methods

### Cultivation of HepG2 cells and preparation of HepG2 spheroids

Human liver hepatocellular carcinoma (HepG2) cells were provided by SINTEF, Norway. 2D- and 3D-cultured HepG2 cells were grown in low-glucose Dulbecco’s modified Eagles m medium, supplemented with 10% foetal bovine serum (FBS) and 1% penicillin–streptomycin (Gibco, Invitrogen Ltd, VWR) and maintained at 37 °C and 5% CO_2_. For all experiments, HepG2 cell passage number was restricted between ten and twenty. At 80% confluence, cells were detached from T75 flasks (Thermo Fisher, Ireland) using TryplE^™^ (Gibco, Invitrogen, Oregon, USA), centrifuged and resuspended in 1-ml culture medium. Cells were counted using a Countess automated cell counter (Thermo Fisher, Ireland) and seeded in either 2D or 3D environments in an appropriate manner for the experimental endpoint under investigation.

**Fig. 1:**
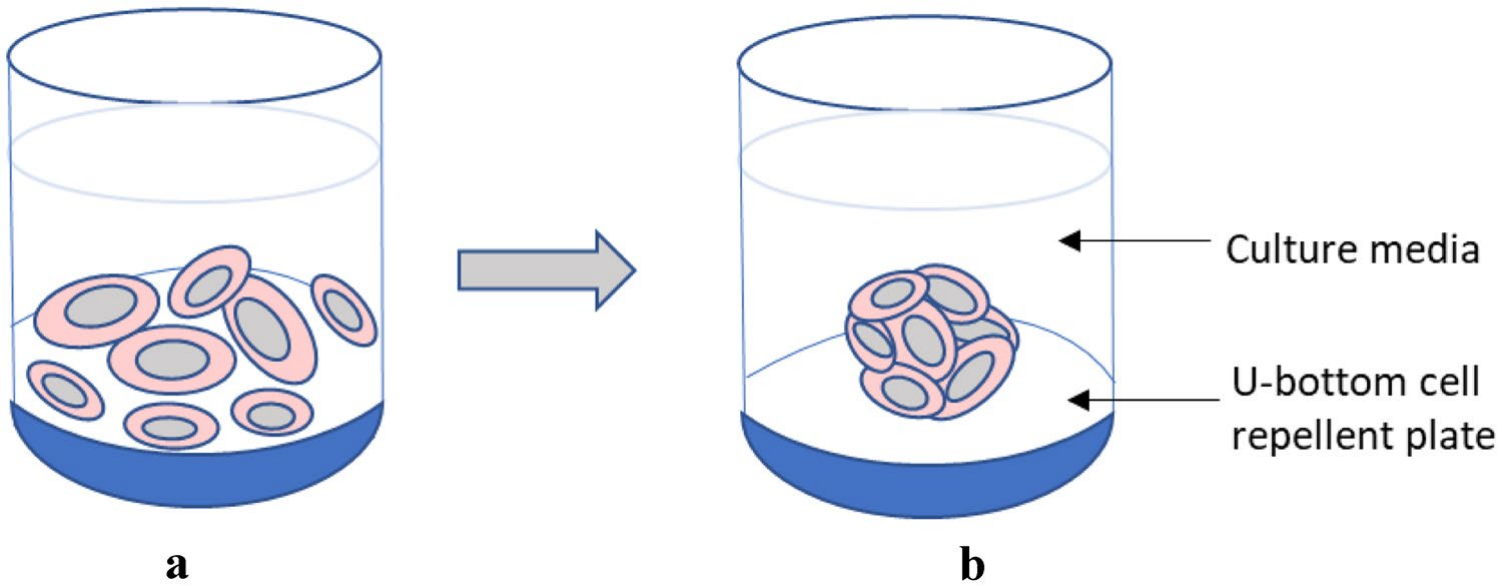
Schematic of HepG2 spheroid formation in CellStar^®^ cell-repellent plates. **A** Single cells are seeded into non-adherent round bottom wells. **B** After 6 h, cells cluster together to form cell spheroids

For 2D monolayers, 10,000 HepG2 cells were seeded on 96-well plates (Thermo Fisher, Ireland). Cells were left to adhere for 24 h before being treated with chosen NBMs for desired timepoints. For HepG2 spheroid formation, cells were seeded in CellStar^®^ 96 well ultra-low attachment (ULA) cell-repellent plates (Grenier, BioOne, UK) at a density of 1000 cells per well in 100 µl medium (Fig. 1). After 3 days of incubation at 37 °C with 5% CO_2_, medium was changed with care taken not to disturb the spheroids. After 1 week, spheroids had an approximate diameter of 300 µm and were taken for further analysis or exposed to NBMs (both detailed below).

### NBM preparation

Five NBMs were used in this study: 20 nm PEGylated AuNP (purchased from nanoComposix, San Diego, California); LipImage^™^ 815, a nanostructured lipid carrier (NLC) encapsulating IR780 dye (kindly supplied by CEA-LETI, France); three polymeric NPs all provided by SINTEF (Trondheim, Norway), i.e. PACA, an unloaded polymeric nanoparticle, PACA loaded with NR668-dye and Cabazitaxel (CBZ) loaded PACA. A nanoparticle control, 10 µg/ml TiO_2_ (NM101 supplied by JRC, Italy), in both static and dynamic conditions, was also included in the study design. A concentration of 10 µg/ml was chosen as control as it kills < 50% cells.

The AuNP were supplied at a mass concentration of 1.00 mg/ml and a molar particle concentration of 2.5 × 10^8^ particles (mol/l). Particles were 20 nm in size. Dispersion medium used was milliQ water. The AuNP were deemed sterile and endotoxin free, i.e. BioPure^™^, with an endotoxin quantity of < 5 EU/ml (within acceptance criteria). AuNP were negatively charged, with a zeta potential of −24 mV. LipImage^™^ 815 was provided at a particle concentration of 95 mg/ml (9.5%) and a dye concentration (HPLC) of 239.5 µM (252 µM/100 mg particle). LipImage^™^ 815 has a diameter of 80 nm. Dispersion medium used was 154 mM NaCl and ascorbic acid (1.75 g/l). LipImage^™^ 815 was deemed sterile and endotoxin free, with an endotoxin quantity of < 1EU/ml (within acceptable amount).

PACA NPs were synthesised under aseptic conditions at SINTEF by mini-emulsion polymerisation. Prior to synthesis, all solutions were sterile filtered, and all equipment was autoclaved. An oil phase consisting of poly(ethylbutyl cyanoacrylate) (PEBCA) (Cuantum Medical Cosmetics) containing 2 wt% Miglyol 812 (Cremer) and 10 wt% vanillin was prepared. For drug-loaded particles, the oil phase was added 12 wt% cabazitaxel (BioChemPartner) and only 2 wt% vanillin was used. For dye-loaded particles, the oil phase was added NR668 (modified Nile Red), custom synthesised at SINTEF [22]. The oil phase was then added to an aqueous phase consisting of 0.1 M HCl containing the two PEG stabilisers (Brij^®^L23 and Kolliphor^®^HS15, both Sigma-Aldrich, 5 wt% of each). The water and oil phases were mixed and immediately sonicated for 3 min on ice (6 × 30 s intervals, 60% amplitude, Branson Ultrasonics digital sonifier). The solution was rotated (15 rpm) at room temperature overnight. The pH was then adjusted to 5.0 to allow further polymerisation for 5 h at room temperature. The dispersions were dialysed (Spectra/Por dialysis membrane MWCO 100.000 Da) against 1 mM HCl to remove unreacted PEG. The size (z-average), polydispersity index (PDI) and the zeta potential of the NPs in phosphate buffer (10 mM, pH 7.0) were measured by dynamic light scattering (DLS) and laser Doppler micro-electrophoresis using a Zetasizer Nano ZS (Malvern Instruments). To calculate the amount of encapsulated drug, the drug was extracted from the particles by dissolving them in acetone (1:10) and quantified by liquid chromatography coupled to mass spectrometry (LC–MS/MS) using an Agilent 1290 HPLC system coupled to an Agilent 6490 triple quadrupole mass spectrometer.

### Hydrodynamic diameter and zeta potential measurements of the NPs

All samples were stored at 4 °C and equilibrated to RT before characterisation, which was kindly undertaken in house by Gabriele Vella, LBCAM, TCD. For nanoparticle tracking analysis (NTA), the NS500 NanoSight (Malvern Panalytical, UK) along with the Nanosight 3.2 software package (NTA build 3.2.16) following the European Union Nanomedicine Characterisation Laboratory (EUNCL) approved protocol was used [23]. NBMs were prepared and diluted between 1:5000 and 1:100,000 using Dulbecco’s phosphate-buffered saline (D-PBS) buffer (-MgCl_2_ and CaCl_2_). A 405-nm laser was used to visualise particles present in a given field of view. Sixty-second recordings of the laser interacting with particles are captured using an EM-CCD camera. The camera level and focus were manually controlled and chosen by the operator (camera level = 10 for the 1:5,000 and 1:10,000 dilutions; camera level = 13 for the 1:100,000 dilution). The detection level was chosen by the operator (detection level = 3 in all dilutions) and the recordings were subsequently analysed by the Nanosight 3.2 software to determine particle numbers per frame and sample concentrations. Through the phenomenon of Brownian motion, the particle size can be determined by the software. The D-PBS used in the dilution of NBMs was also analysed to assess background particle levels. Thirty-nanometre gold citrate NPs of known size were used as reference materials for the Nanosight.

For dynamic light scattering (DLS), NBMs were analysed using a Zetasizer Nano ZS system (Malvern UK), running Zetasizer version 7.13, using the EUNCL approved -PCC-001 SOP ‘Measuring Batch Mode DLS’ [23]. Of the sample for DLS, 1:100 dilution was made up in D-PBS buffer (-MgCl_2_ and CaCl_2_). Samples were pipetted to ensure adequate mixing. Samples were loaded into a DTS0012 disposable cuvette and were subjected to a 300-s equilibration time as per the EUNCL SOP. A total of twelve × 10-s runs per measurement were recorded for the sample and were subjected to 10 measurements with a zero second delay between measurements. The backscatter angle (173° NIBS Default) was used in the analysis, with optimum positioning enabled. Automatic attenuation selection was enabled, and the general purpose analysis mode was chosen.

### Morphological assessment of HepG2 spheroids

Brightfield microscopy was used to assess the growth, morphology and size of hepatic spheroids across a month-long culture period. Spheroids were imaged using an epifluorescence microscope (Nikon TE300, equipped with 10X objective and QCapture Software QImaging). All image processing and measurements were undertaken using ImageJ software.

Histological analysis was used to assess the internal structure of hepatic spheroids. Cultures were washed with PBS and fixed for 1 h in 4% paraformaldehyde (PFA), before being embedded in 2% electroendosmosis (EEO) agarose in PBS in plastic moulds. Agarose blocks were dehydrated in increasing concentrations of EtOH (20%, 40%, 60% EtOH in H_2_O), and paraffin-embedded using a tissue processor with the help of Dr Gavin McManus (Trinity Biomedical Sciences Institute, Trinity College Dublin). Paraffin blocks were sectioned using a microtome and sections stained with haematoxylin and eosin (H&E). Briefly, spheroid section slides were de-waxed and rehydrated by passing them through solvents in the following manner: xylene 5 min, xylene 4 min, 99% industrial methylated spirits (IMS) 3 min, 90% IMS 2 min, 70% IMS 1 min, before running them in cold water for 1 min. Slides were then placed in haematoxylin for 5 min before being rinsed in running tap water for 5 min. Slides were dipped in acid alcohol (1%) for 2 s and rinsed in tap water for 30 s, before staining with eosin (5 min). Slides were briefly rinsed in tap water again, with care taken to not remove eosin. Sections were then dehydrated and cleared in the following manner: 70% IMS 1 min, 90% IMS 1 min, 99% IMS, 2 min, xylene 3 min, xylene 3 min. Slides were mounted using DPX mounting media (comprised of distyrene, a plasticiser and xylene), with cover slide added at a 45° angle. Stained tissue sections were then imaged by epifluorescence microscopy. Image analysis was performed by ImageJ software.

### Quantification of cell number in spheroids

The total cell number in HepG2 spheroids was determined by adding TryplE^™^ to spheroids (3 pooled spheroids) and mechanically dissociating by vigorous pipetting, staining with 1:1 trypan blue and counting cells using the Countess^™^ automated cell counter (Thermo Fisher, Ireland).

### Albumin quantification

Release of human serum albumin was quantified using R&D DuoSet Human Serum Albumin ELISA kit (R&D Systems, Ireland), carried out according to the manufacturer’s protocol on supernatants collected from cell models. Albumin content in supernatants was extrapolated and quantified from an albumin standard curve included in the experimental design.

### Sensitivity to hepatotoxins

Sensitivity of spheroid cultures to a panel of common hepatotoxins was tested using a protocol detailed previously in works from Gaskell et al. [24] and Bell et al. [25] and using information listed in the MIP-DILI training set [26]. The hepatotoxins included acetaminophen, diclofenac, trovafloxacin and fialuridine. HepG2 spheroids grown for 3 days and 10 days were exposed to the hepatotoxins, diluted in cell culture medium, for 4 days, with an addition dose administered 2 days after the first exposure. Concentrations tested for each compound were the following:

1. Acetaminophen**:** 10000, 5000, 1250, 625, 300, 150, 75 µg/ml
2. Diclofenac: 400, 200, 100, 50, 25, 12.5, 6.25 µg/ml
3. Trovafloxacin: 500, 200, 100, 50, 25, 12.5, 6.25 µg/ml
4. Fialuridine: 200, 100, 50, 25, 12.5, 6.25, 3.15 µg/ml

Viability of the treated cultures was determined using the CellTiter-Glo^®^ assay (Thermo Fisher, Ireland) as per the manufacturer’s protocol. Culture medium alone with CellTiter-Glo^®^ reagent was used as a blank control and subtracted from all sample values. Cell viability was calculated as a percentage of untreated vehicle control samples. A dose-dependent response curve was plotted, and estimations were made about what concentration of drug would cause a 50% reduction in cell viability (IC50 value). Experiments were run in triplicate (*n* = 3).

### Exposure to NBMs

2D and 3D cultures were exposed to AuNP, LipImage^™^ 815, or various PACAs, using a variety of concentrations detailed below in Table 1. The rationale behind the experimental design and the concentrations tested came from extensive dialogue and work we have undertaken within the REFINE project [27] and published degree thesis of Tutty, MA [28].

**Table 1:** NBMs used in this study and the concentrations used

**NBM**	**Exposure (µg/ml; 30 min, 3 h, 24 h)**
AuNP	1, 5, 10, 20, 30, 50
LipImage^™^ 815	10, 50, 100, 200, 500, 1000
PACA	1, 5, 10, 20, 32, 64, 128, 256, 512, 1024
CBZ-loaded PACA	32, 64, 128, 256, 512, 1024
NR668-loaded PACA	32, 64, 128, 256, 512, 1024

Stock NBM suspensions were vortexed and appropriate dilutions made in cell culture medium. For NBM exposures, briefly, cell culture medium was removed from the wells of culture plates and wells washed with pre-warmed PBS. For adenosine 5′-triphosphate (ATP) quantification and lactate dehydrogenase (LDH) assay, 100 µl of each NBM concentration diluted in culture medium was added to each well for three incubation/exposure timepoints, 24, 48 and 72 h.

### Viability and cytotoxicity assessment via CellTiter-Glo^®^ and LDH assays

**Fig. 2:**
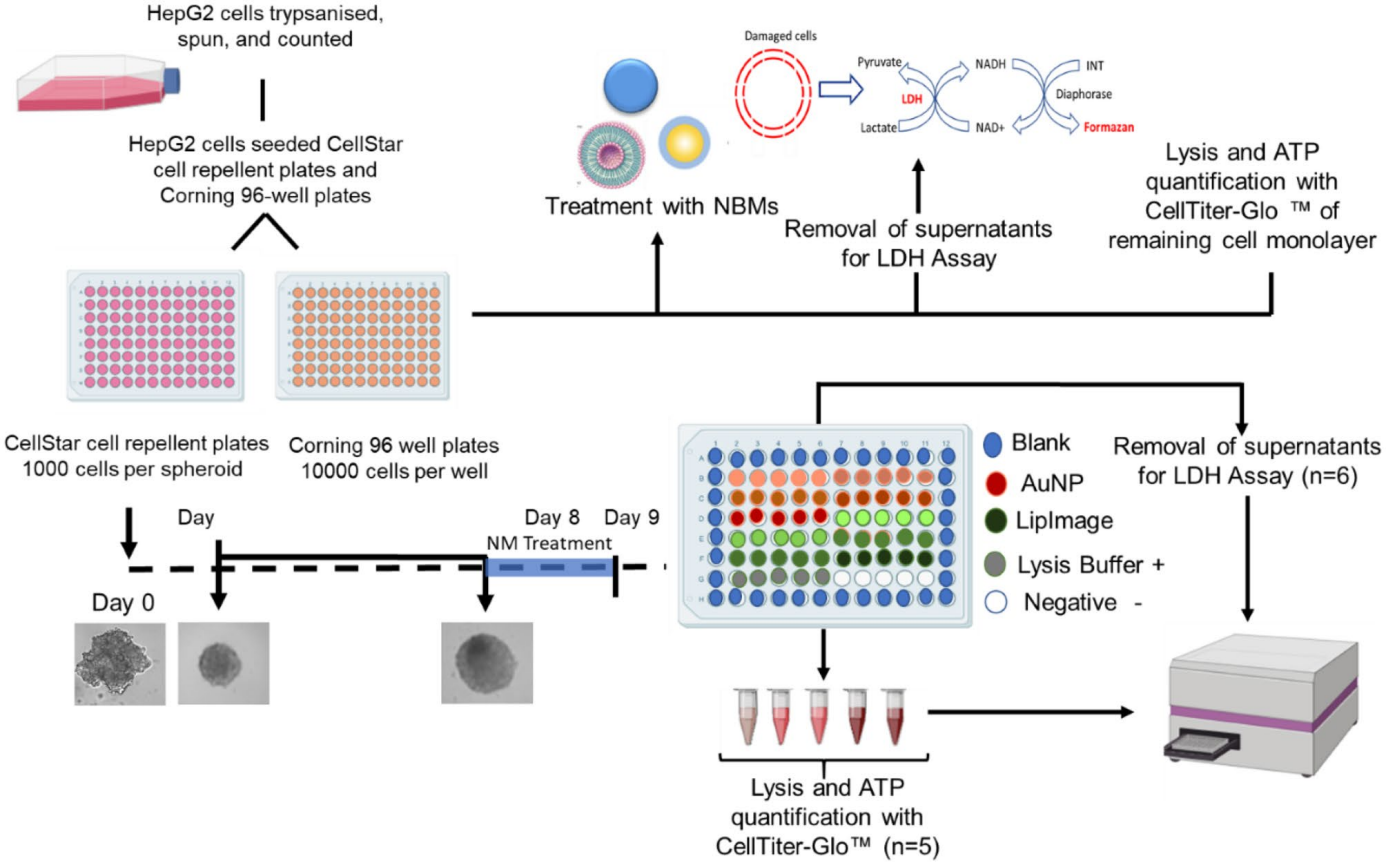
Experimental workflow for assessing NBM hepatotoxicity in vitro by measuring changes in viability and cytotoxicity following multiple endpoint exposure

Experimental workflow for viability and cytotoxicity assays is detailed in Fig. 2.

The CellTiter-Glo^®^ Luminescent Cell Viability Assay is a method of assessing cell viability in cultures based on the quantitation of ATP present, an indicator of metabolically active cells. Cultures were washed with pre-warmed PBS and ATP content quantified using CellTiter-Glo^®^ (Promega Corporation, MyBio Ltd, Ireland), performed as per the manufacturer’s protocol. All samples were tested in triplicate (*n*_replicates_ = 3), and experiments were repeated three times (*n*_tests_ = 3). Data are presented as average ± standard deviation of the mean. Assays and experimental controls were included in the experimental design, including untreated controls of culture medium alone and no NBMs. Experimental positive control, at 125 µM valinomycin, was chosen as this concentration caused < 50% maximal cell death after 24 h. Assay positive control was 10 × lysis buffer, as from the supplier. NBM positive control was 10 µg/ml TiO_2_ as this concentration caused < 50% maximal cell death after 24 h when studied in both static and dynamic conditions, as shown in Fig. 15.

The LDH assay is a cytotoxicity assay which is used to assess the level of plasma membrane damage in a cell population. Supernatants were harvested from 2D cultures/3D monoculture and multicellular spheroids exposed to NBMs at three different incubation/exposure timepoints, 24, 48 and 72 h. LDH release in the supernatants (an indirect measure of cytotoxicity responses) was assessed using the Thermo Scientific Pierce LDH Cytotoxicity Assay Kit (Fisher Scientific, Ireland), following the manufacturers’ protocols. Untreated cultures and 2D cultures/3D spheroids exposed to LDH lysis buffer (1 × in supplemented culture medium; 45 min; 37 °C) were included in the experimental design as negative (NT) and positive (PT) controls, respectively. Absorbance values at wavelengths (λ) equal to 490 and 680 nm were read using an Epoch microplate reader (Biotek, Mason Technologies, Ireland). The LDH activity was calculated using Eq. (1), whereas the percentage (%) cytotoxicity was extrapolated from Eq. (2).

1 $$L{\varvec{DH}}\ activity=\mathrm{Absorbance}\ \lambda =490\ \mathrm{nm}-\mathrm{Absorbance}\ \lambda =680\ \mathrm{nm}$$

2 $${\varvec{Cytotoxicity}}\ \left(\%\right)=\frac{\mathrm{LDH\ activity\ Treated\ culture}-\mathrm{LDH\ activity\ }\ \left(\mathrm{NT}\right)}{\mathrm{LDH\ activity\ PT}-\mathrm{LDH\ activity}\ \left(\mathrm{NT}\right)}$$

All samples were tested in triplicate (*n*_*r*eplicates_ = 3), and experiments were repeated three times (*n*_tests_ = 3). Data are presented as average ± standard error of the mean (SEM).

To determine if any variation in the experimental conditions, i.e. static vs dynamic, had any effect on the final cytotoxicity endpoints, positive and negative controls were introduced in the experimental design to measure the LDH assay and experiment accuracy. Positive control for nanoparticle cytotoxicity was a titanium dioxide (TiO_2_) nanoparticle used at EC50 concentration of 10 µg/ml. Reagent positive control (PT) was adopted in valinomycin at a concentration of 125 µM, whereas assay positive control was 50 µl 10 × lysis buffer provided with LDH kit. Conversely, the negative controls (NT) included were cell culture medium alone (as vehicle or diluent) and plates in incubator as per normal culture conditions (as static condition), and dynamic condition is reflected by the use of a Mimetas rocker.

### Statistical analysis

Results are presented as mean with standard deviation (SD) of 3 independent experiments (*n* = 3), unless otherwise mentioned. Statistical analysis was performed by two-way ANOVA, with post hoc Sidak test. All graphs and statistical analysis were undertaken using GraphPad Prism 9, Version 9.4.0 (GraphPad Software, Inc, San Diego, USA). *p* values are marked by * as *p* < 0.05, ** as *p* < 0.01, *** as *p* < 0.001 and **** as *p* < 0.0001.

## Results

### Characterisation of NPs

The quantitative analysis of the physicochemical characteristics of any NBM is an essential step in order to select a suitable formulation to bring forward in any study. In order to determine if NBMs had remained stable during transit, NBM characterisation was undertaken for the AuNP, LipImage^™^ 815 and PACA NBMs prior to any further experimentation, with results compared to characterisation data from supplier.

#### Hydrodynamic diameter and zeta potential

It is widely known that both size and shape greatly influence NBM-induced toxicity. The potential of an NBM formulation to induce cellular damage is directly dependent on its size. Therefore, each nano-formulation used in this study was carefully characterised within the LBCAM using conventional nano-characterisation techniques and validated protocols, with all data compared to supplied manufacturer’s data.

**Fig. 3:**
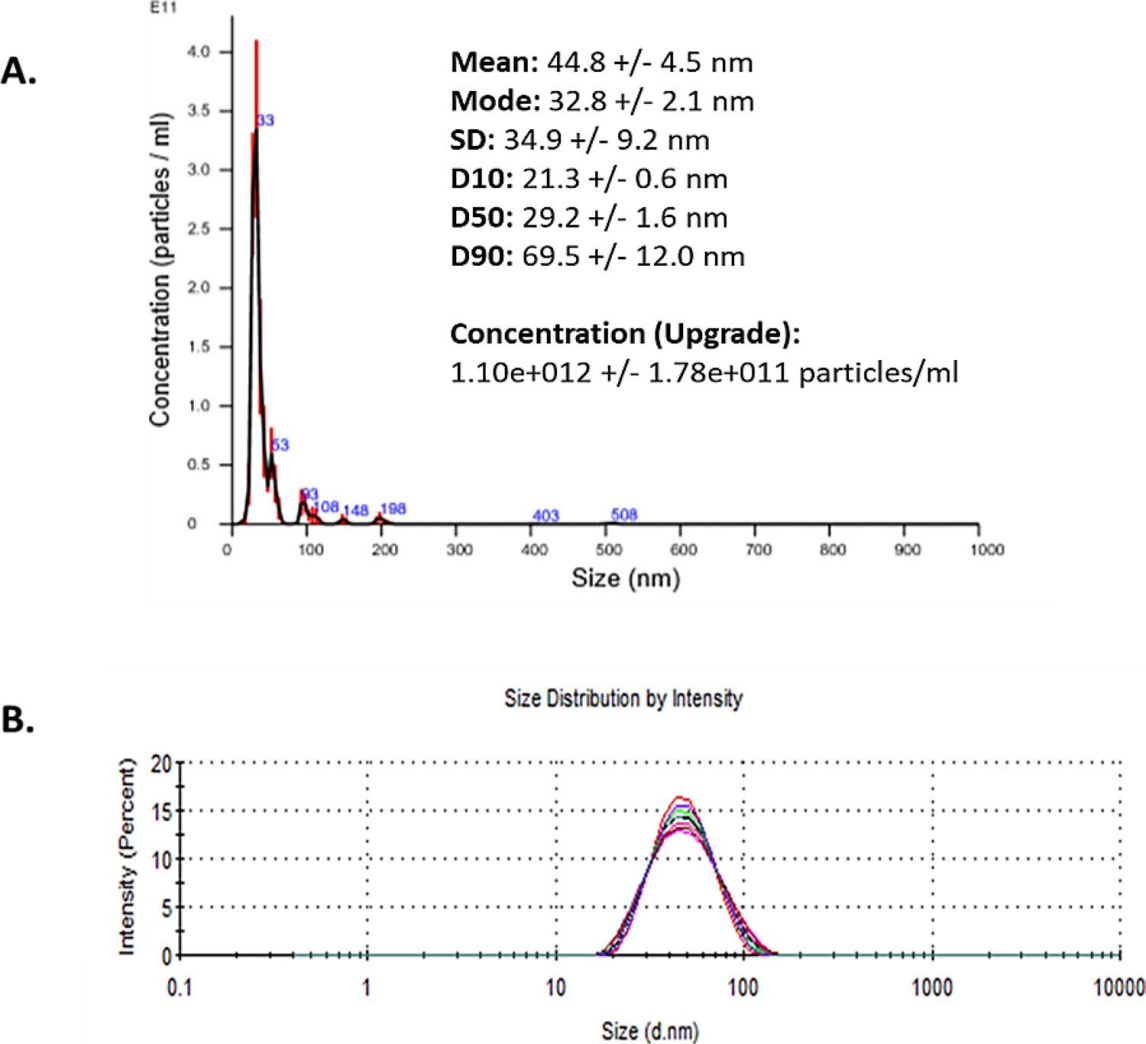
NTA and DLS analysis of AuNP. **A** Nanoparticle tracking analysis (NTA) size versus concentration graph. AuNPs were diluted to 10 µg/ml in particle-free water (Sigma-Aldrich, Ireland) and analysed through five × 60-s recordings. **B** Dynamic light scattering (DLS) graphs depicting size distribution

The AuNP used in this study were provided at a mass concentration of 1.00 mg/ml and a molar particle concentration of 2.5 × 10^8^ particles (mol/l) in MilliQ H_2_O. In line with the EUNCL developed protocols for NBM size and concentration characterisation, analysis was carried out within the LBCAM and compared to supplied data (Table 2). NTA and DLS plots are reported in Fig. 3. NTA reported a mean size of 43.4 nm, which closely resembles the DLS measurements of 42.45 nm. DLS measured an average zeta potential −26.4 mV at pH 7, making the AuNP anionic. Some small aggregates are present at approximately 100 nm. PDI index, obtained from DLS, is 0.102, making AuNP monodispersed. LBCAM characterisation is closely related to supplier values provided.

**Table 2:** Summary and comparison of characterisation data by NTA and DLS for AuNP

**AuNP measured parameter**	**Supplier value**	**TCD value**
Mean hydrodynamic size (DLS)	45.5 nm	42.45 nm
Mean hydrodynamic size (NTA)	Not provided	43.4 nm
Polydispersity index (PDI) (DLS)	Not provided	0.102
Zeta potential	−26 mV	−26.4 mV

**Fig. 4:**
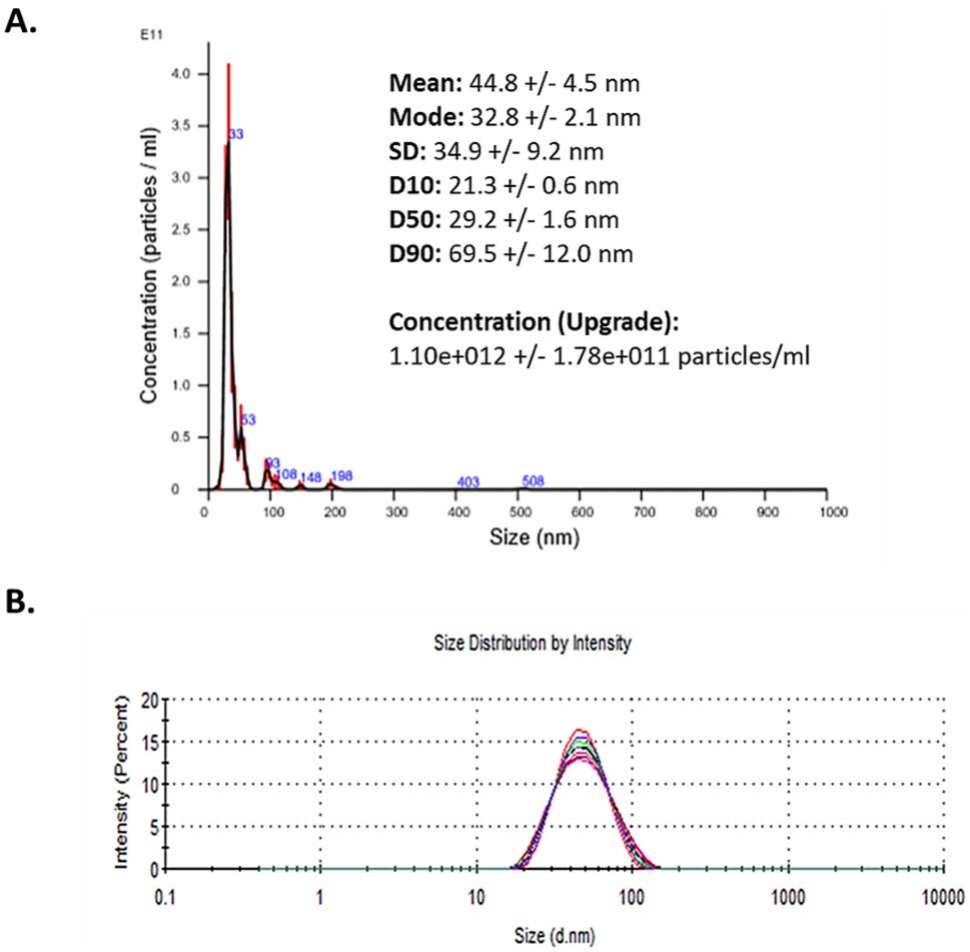
NTA and DLS analysis of LipImage^™^ 815. **A** Nanoparticle tracking analysis (NTA) size versus concentration graph. LipImage^™^ 815 was diluted to 10 µg/ml in particle-free water (Sigma-Aldrich, Ireland) and analysed through five × 60-s recordings. **B** Dynamic light scattering (DLS) graphs depicting size distribution

LipImage^™^ 815 was provided by CEA-LETI (France), at a particle concentration of 95 mg/ml (9.5%) and a dye concentration (HPLC) of 239.5 µM (252 µM/100 mg particle). Dispersion media used were 154 mM NaCl and ascorbic acid (1.75 g/l). In line with the EUNCL developed protocols for NBM size and concentration characterisation, analysis was carried out within the LBCAM and compared to supplied data (Table 3). NTA reported a mean size of 50.72 nm, with DLS measurements reporting a mean size of 72.7 nm. A small aggregate peak is observed at 163 nm in NTA graph. PDI index, obtained from DLS, is 0.11, making LipImage^™^ 815 monodispersed, as showed in Fig. 4. The LBCAM validated characterisation is closely related to supplier values provided.

**Table 3:** Summary and comparison of characterisation data by NTA and DLS for LipImage^™^ 815

**LipImage**	**Supplier value**	**TCD value**
Mean hydrodynamic size (DLS)	52.2 nm	50.72 nm
Mean hydrodynamic size (NTA)	Not provided	72.7 nm
Polydispersity index (PDI) (DLS)	< 0.102	0.11

**Fig. 5:**
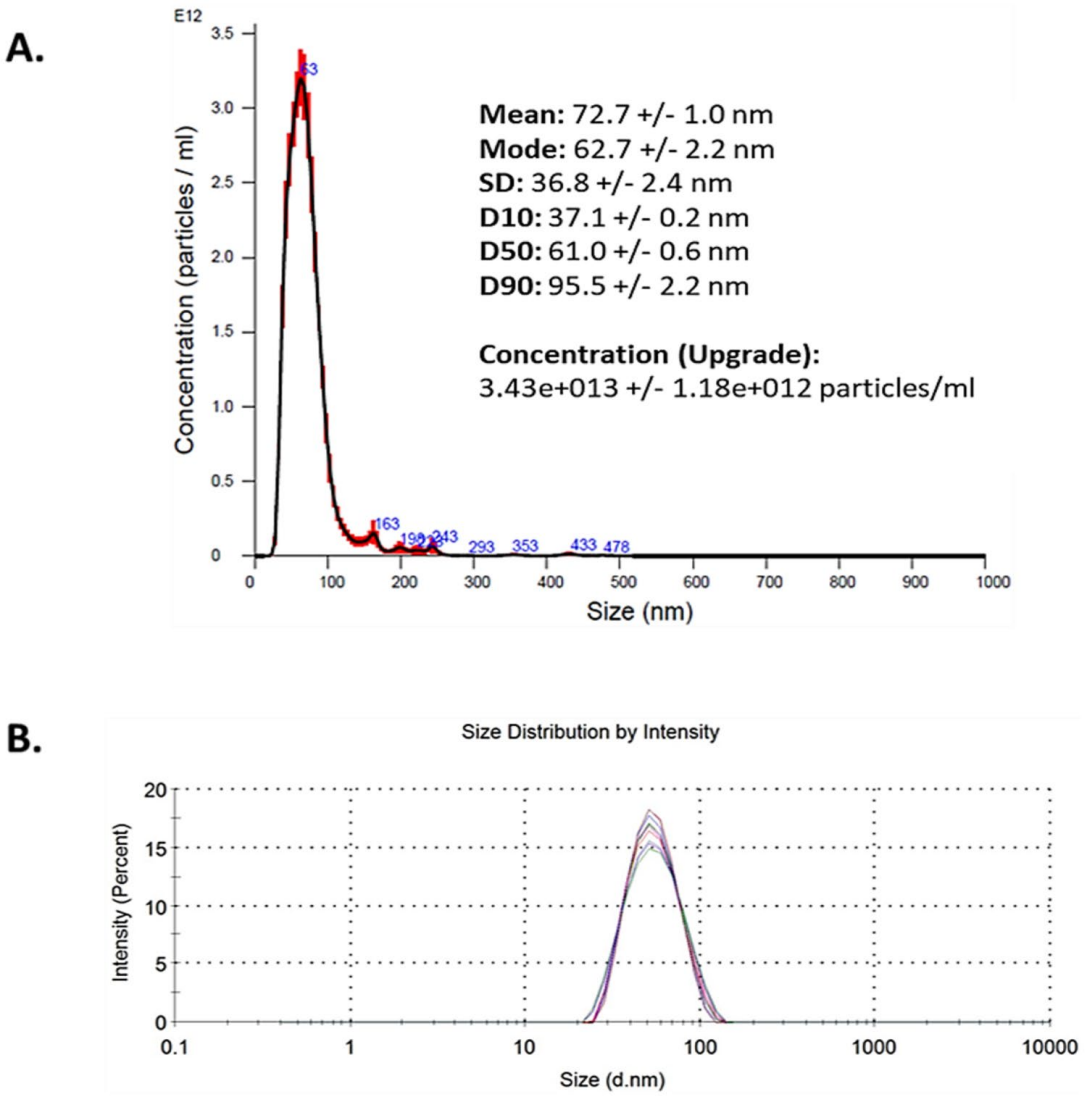
NTA and DLS analysis of unloaded PACA. **A** Nanoparticle tracking analysis size versus concentration graph. PACA was diluted to 10 µg/ml in particle-free water (Sigma-Aldrich, Ireland) and analysed through five × 60-s recordings. **B** Dynamic light scattering graphs depicting size distribution

With regard to unloaded PACA NBMs, comparison between characterisation data supplied and characterisation obtained by the LBCAM is found in Table 4. NTA and DLS plots are reported in Fig. 5. NTA reported a mean size of 94 nm and DLS measurement of 134.8 nm. Suppliers noted an average zeta potential −3.2 mV at pH 7, making PACA neutral. PDI index, obtained from DLS, is 0.092, making PACA monodispersed. LBCAM characterisation is closely related to supplier values provided.

**Table 4:** Summary and comparison of characterisation data by NTA and DLS for unloaded PACA

**PACA measured parameter**	**Supplier value**	**TCD value**
Mean hydrodynamic size (DLS)	134	134.8
Mean hydrodynamic size (NTA)	Not provided	94
Polydispersity index (PDI) (DLS)	0.11	0.092
Zeta potential	−3.2 mV	Not provided

**Fig. 6:**
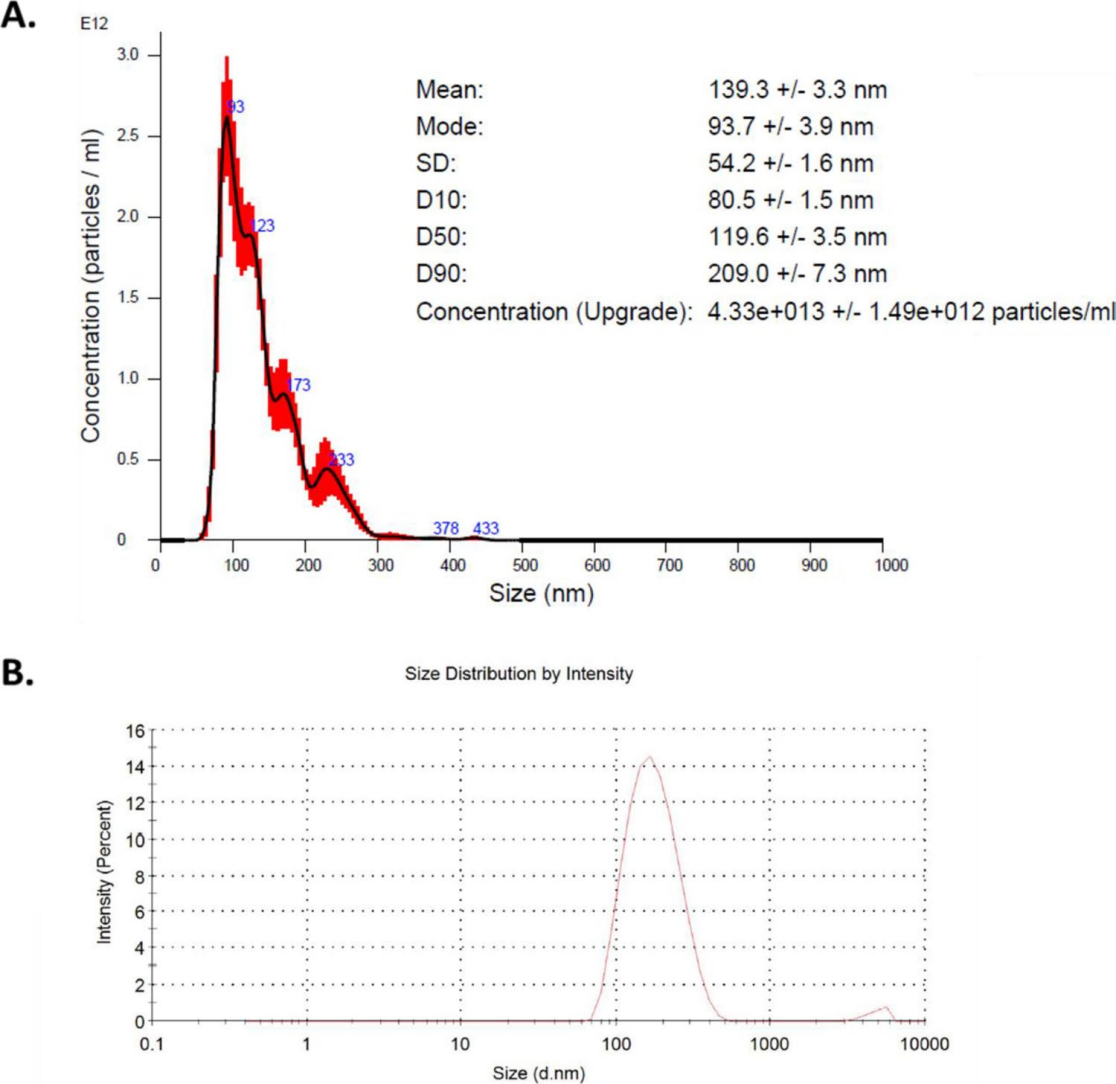
NTA and DLS analysis of NR668-loaded PACA. **A** Nanoparticle tracking analysis size versus concentration graph. PACA was diluted to 10 µg/ml in particle-free water (Sigma-Aldrich, Ireland) and analysed through five × 60-s recordings. **B** Dynamic light scattering graphs depicting size distribution

The NR668-loaded PACA NBM was provided at a stock particle concentration of 105 mg NP/ml. Dispersion medium used was 1 mM HCl in sterile distilled water. Analysis was carried out within the LBCAM using validated protocols and compared to supplied data (Table 5), with NTA reporting a mean size of 140 nm and DLS measurement of 164.7 nm. Please note that from the supplier DLS analysis, it showed a measured average zeta potential of −3.6 mV at pH 7, making this PACA NBM neutral. PDI index, obtained from DLS, is 0.18, making PACA monodispersed. LBCAM characterisation is closely related to the supplier values provided (Fig. 6).

**Table 5:** Summary and comparison of characterisation data by NTA and DLS for NR668 PACA

**NR668-PACA measured parameter**	**Supplier value**	**TCD value**
Mean hydrodynamic size (DLS)	178	164.7
Mean hydrodynamic size (NTA)	Not provided	140
Polydispersity index (PDI) (DLS)	0.28	0.18
Zeta potential	−3.6 mV	Not provided

**Fig. 7:**
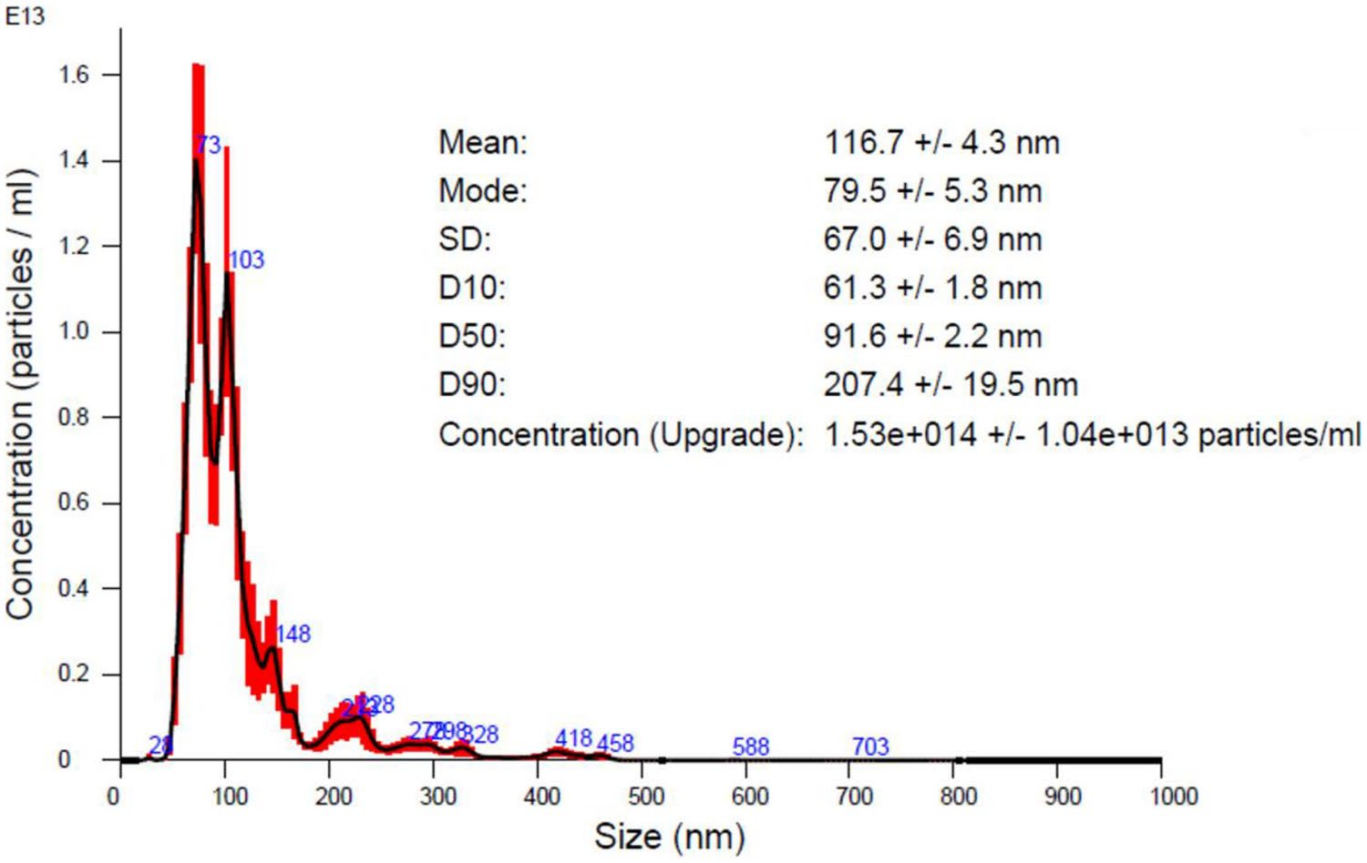
NTA analysis of CBZ-loaded PACA. Nanoparticle tracking analysis size versus concentration graph. CBZ-PACA were diluted to 10 µg/ml in particle-free water (Sigma-Aldrich, Ireland) and analysed through five × 60-s recordings

The final NBM, CBZ-loaded PACA was provided at a particle concentration of 107 mg/ml and with a drug loading concentration of 10.8 mg/ml (wt% or particle weight as measured by mass spectrometry (MS) and 12.9 mg/ml drug concentration in stock, also measured by liquid chromatography mass spectrometry (LC–MS)). Characterisation undertaken within LBCAM is detailed in Table 6. NTA reported a mean size of 116.7 nm (Fig. 7). DLS measurement provided by supplier was measured at 121.8 nm and an average zeta potential −5.5 mV at pH 7, making this PACA NBM neutral. PDI index, obtained from DLS, is 0.14.

**Table 6:** Summary and comparison of characterisation data by NTA and DLS for CBZ-PACA

**CBZ-PACA measured parameter**	**Supplier value**	**TCD value**
Mean hydrodynamic size (DLS)	121.8	N/A
Mean hydrodynamic size (NTA)	Not provided	116.7
Polydispersity index (PDI) (DLS)	0.14	N/A
Zeta potential	−5.5 mV	N/A

### Brightfield microscopy and H&E analysis of spheroids

**Fig. 8:**
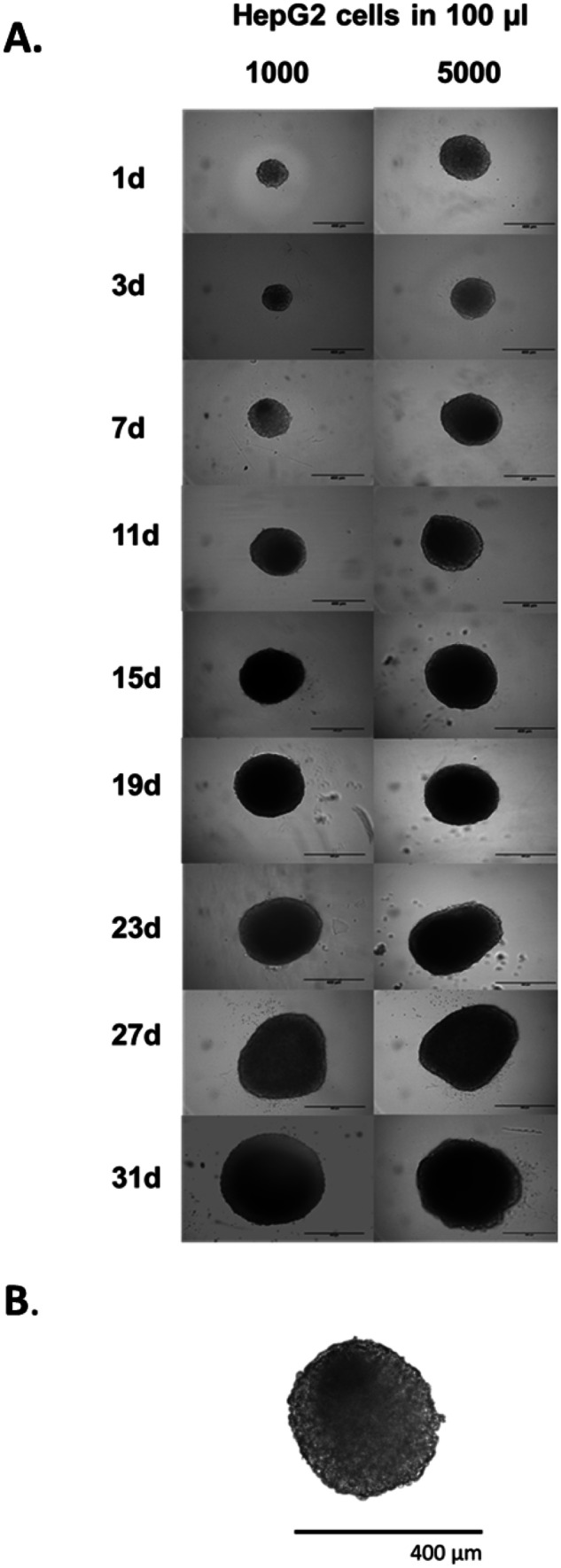
**A** Brightfield image of a HepG2 spheroid cultured up to 31 days. **B** After 7 days, spheroids had defined perimeters, limited cell death and compact structure

Spheroid diameter gradually increased over the culture period, with spheroids appearing generally spherical and uniform in shape (Fig. 8A). Loose spheroids were formed after 1 day, which further compacted to form fully intact spheroids after 3 days. Loss of circular morphology was observed after 19 days. A seeding concentration of 1,000 cells per 100 µl culture medium yielded uniform, circular spheroids, with consistent morphology over time and a diameter of 294.26 ± 10.43 µm at 3 days and of 647.95 ± 16.86 µm at 31 days. At 7 days, the chosen timepoint for any further experiments, spheroids had defined perimeters, limited cell death and compact structure (Fig. 8B).

**Fig. 9:**
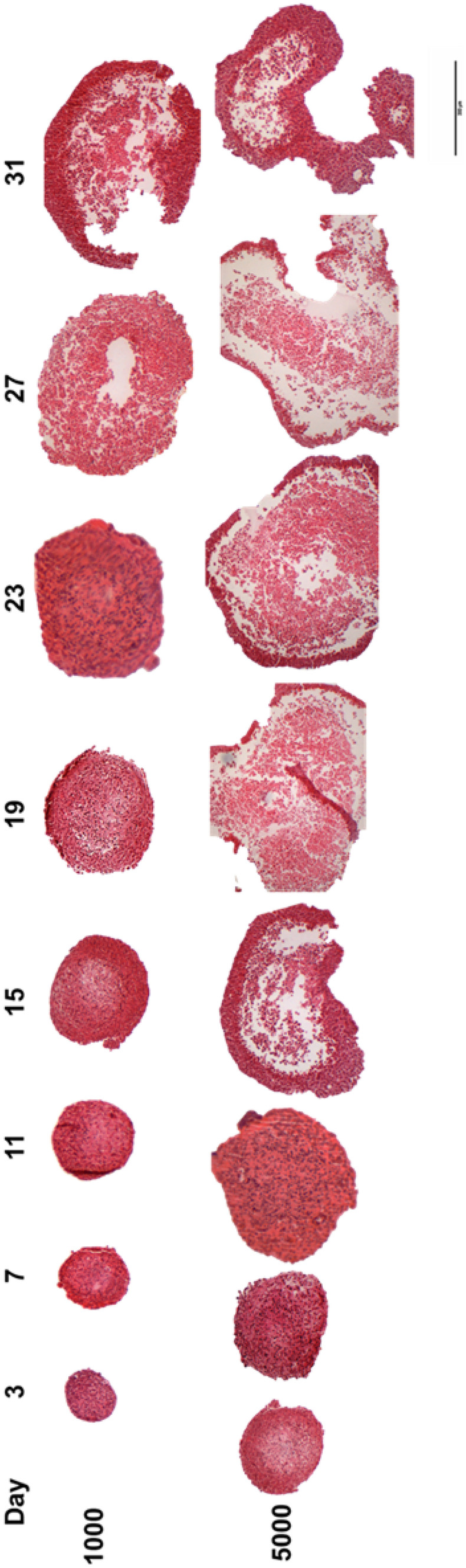
Internal spheroid structure and morphology. Spheroids were created using CellStar^®^ cell-repellent plates from 1000 and 5000 HepG2 cells in 100-µl cell culture medium. Spheroids were fixed in PFA after 3, 7, 11 15, 19, 23, 27 and 31 days in culture, before being suspended in agarose, dehydrated in increasing concentrations of ethanol, paraffin-embedded, sectioned and stained with H&E. Images are representative of the specimens and show mid-sections of the spheroids. Scale bar = 300 µm

To assess changes in internal spheroid structure and in morphology, HepG2 spheroids were paraffin-embedded, sectioned and stained with H&E. Spheroids formed from a cell seeding concentration of 1,000 and 5000 cells per 100-µl culture medium confirmed a compact, uniform internal structure with defined outer perimeters, and direct cell–cell contacts similar to those observed in human liver. Limited apoptosis and no necrotic core are visible up to 27 days when a small patch of necrosis could be observed in spheroid core. At 7 days, the chosen timepoint for any further experiments, spheroids appeared healthy, with no visible cell death observed (Fig. 9).

### Cell count in HepG2 spheroids

**Fig. 10:**
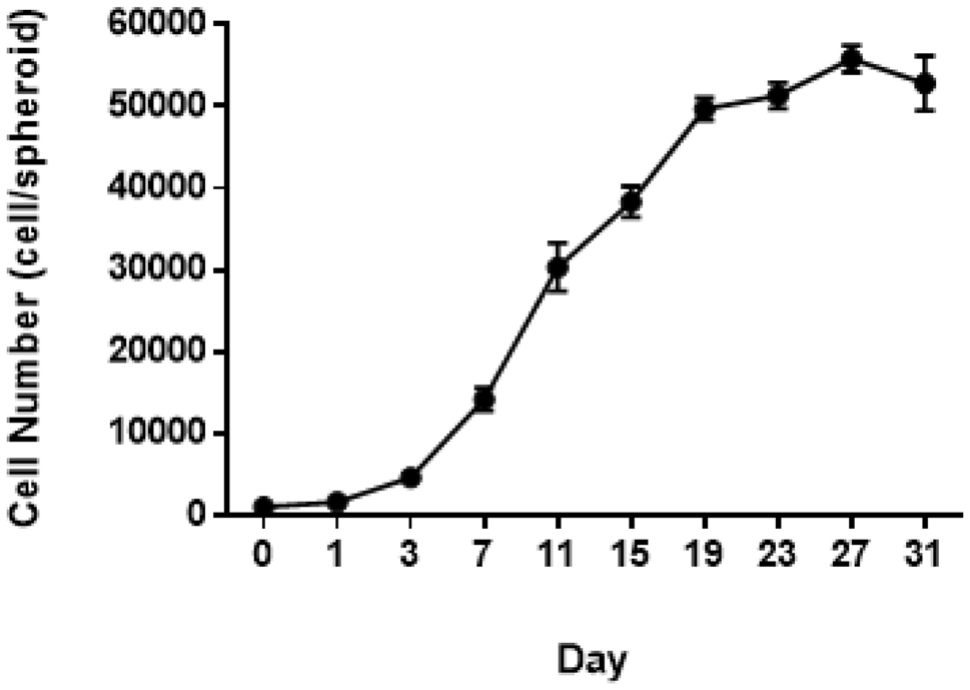
Cell number in HepG2 spheroids grown in CellStar^®^ cell-repellent plates. Spheroids were formed starting from a seeding concentration of 1,000 HepG2 cells per 100-µl culture medium and cultured for 31 days. Data shown as mean ± SEM (*n*_replicates_ = 6)

The increase in spheroid diameter, observed from bright field microscopy, was reflected in an increased growth, reported as cell number over a 1-month period (Fig. 10). The cell number increased as culture time progressed, with a sigmoidal pattern observed. As determined from H&E staining (Fig. 9), no notable signs of nutrient or oxygen shortage, or core necrosis formation, were observed within spheroids, leading to the belief that the plateau reached after approximately 19 days occurs due to reduced cell proliferation and senescence of HepG2 cells within the spheroids.

### Quantification of human serum albumin using ELISA

**Fig. 11:**
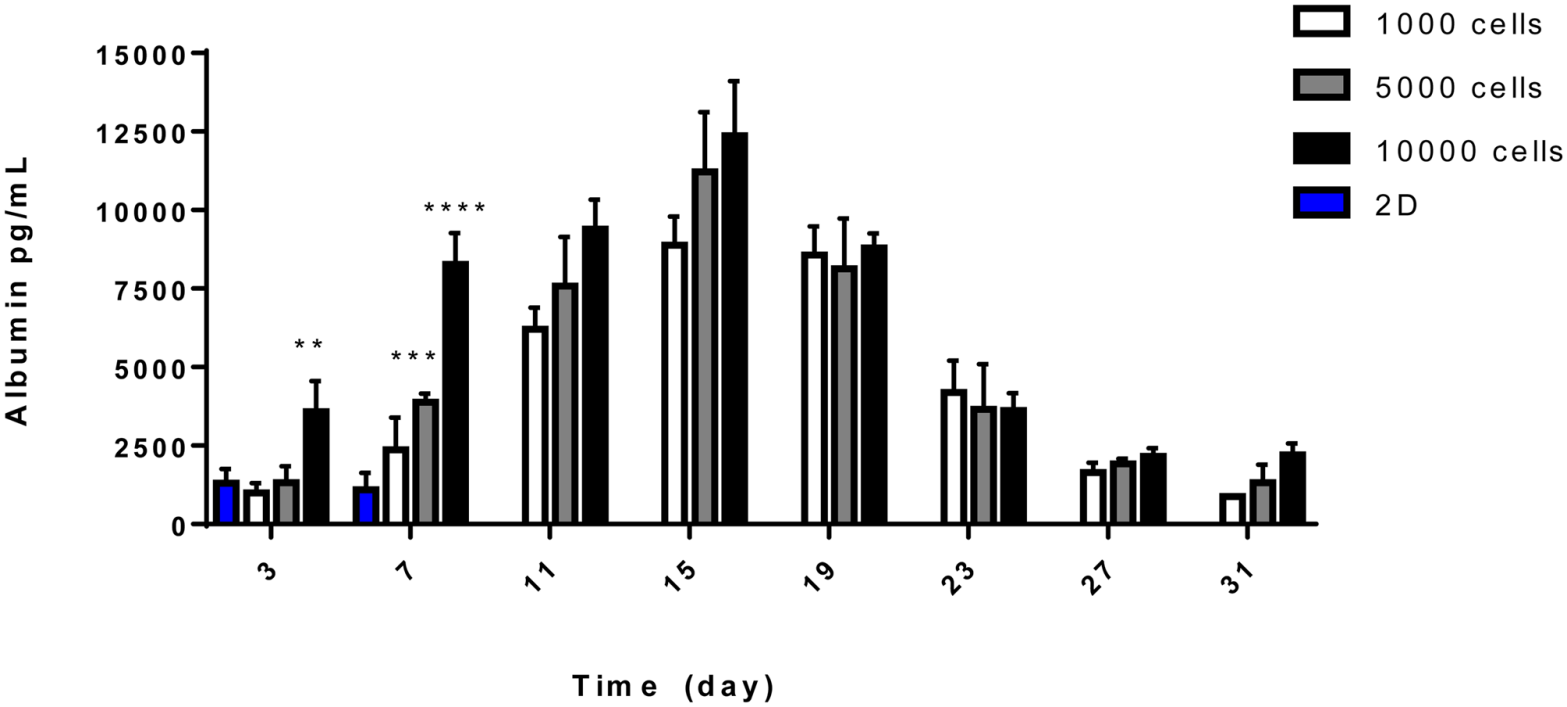
Changes in albumin release from HepG2 spheroids and 2D monolayers overtime. Supernatants from 2D HepG2 cultures and 3D spheroids were collected and analysed over the course of 31 days. HepG2 spheroids were grown starting from a seeding concentration of 1,000 (white bars), 5,000 (grey bars) and 10,000 (black bars) cells per 100-µl cell culture medium. 2D monolayers (blue bars) were formed by seeding 10,000 per 100-µl culture medium. Data represented as mean ± SEM, two-way ANOVA, ** *p* 0.0030, *** *p* 0.0005 and **** *p* < 0.0001 (*n*_replicates_ = 3 in triplicate)

Albumin secretion is often used to assess liver-specific function in spheroids [24, 29–31]. The production of albumin was therefore quantified over 31 days, on supernatants collected from spheroids. Albumin secretion increased over the culture period up to its maximum cumulative release at 15 days (Fig. 11), before dropping after 19 days in culture. After 3 days, the albumin secretion of 3D spheroids was comparable to that of 2D monolayers after 3 days, with the exemption of 3D spheroids grown from a cell seeding concentration of 10,000 cells per 100-µl culture medium. Conversely, it was more than doubled after 7 days in culture.

### Ability of spheroids to detect hepatotoxicity–sensitivity of HepG2 spheroids to human hepatotoxins and comparison to 2D monolayers

**Fig. 12:**
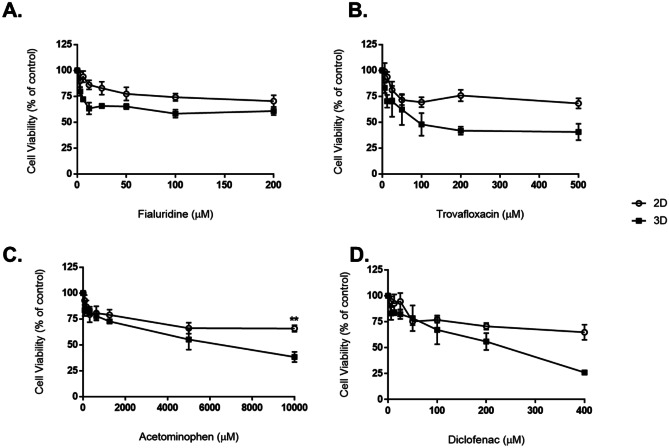
Changes in cell viability in 2D monolayers and 3D spheroids of HepG2 cells following exposure to four common hepatotoxins. Spheroids and 2D monolayers were treated with four hepatotoxic compounds, namely **A** fialuridine, **B** trovafloxacin, **C** acetaminophen and **D** diclofenac. 3D spheroids were exposed using a 4-day repeat-dosing regime, whereby spheroids were treated at day 3 and day 5, before readout was taken on day 7. For 2D monolayers, 24-h acute dosing was used. Cell viability, following treatments, was determined and plotted as a percentage of the untreated control. Data represented as mean ± SEM (*n*_replicates_ = 3). The symbol (**) indicates *p* < 0.01 (multiple *t* tests with Bonferroni Dunn method)

Spheroids and HepG2 monolayers were treated with varying concentrations of four common hepatotoxic compounds, namely acetaminophen, diclofenac, fialuridine and trovafloxacin. Following a protocol detailed in Gaskell et al., a 4-day repeat-dosing regimen was used to expose 3D spheroids, i.e. spheroids were cultured for 3 days before being treated with hepatotoxic compounds for 2 days, before doing is repeated again on day 5. Spheroids then remained in culture untouched until ATP quantification assay was undertaken on day 7. For 2D monolayers, however, acute 24-h dosing only was assessed as the monolayers became over-confluent and exhibited reduced viability after 48 h in culture. After treatment with the hepatotoxic compounds, a dose-dependent reduction in cell viability was observed in spheroids (Fig. 12). Each of the four chosen compounds was more toxic in 3D spheroids than in 2D monocultures, although differences were not statistically significant. The concentration causing a 50% reduction in cell viability (IC_50_) was estimated for each drug tested from the graphs, and is reported in Table 7. A comparison to data extracted from the scientific literature is also reported.

**Table 7:** Sensitivity of HepG2 spheroids and monolayers to a panel of common hepatotoxins. HepG2 cells and spheroids were treated with varying concentrations of common hepatotoxins. Viability was assessed using the CellTiter-Glo ATP quantification assay with readout taken on day 7, and the concentration of drug to cause a 50% reduction in viability was estimated from assay results. PHH denoted primary human hepatocytes

**Compound**	**IC50 value in HepG2 spheroids–4-day repeat dosing (μM)**	**IC50 value in HepG2 2D monolayer–24-h acute dosing (μM)**
**Fialuridine**	> 200	> 200
**Trovafloxacin**	> 100	> 400
**Acetaminophen**	> 8000	> 10000
**Diclofenac**	> 250	> 400

Size of spheroids or their duration in culture was also found to have no influence on their responses to hepatotoxins, with no significant differences found when the same experiment was performed on spheroids cultured for 3 days or 10 days, following repeated dosing at day 5 and day 12 and readout taken on day 14 (Supplementary Fig. 1).

### Viability of 2D and 3D hepatocellular carcinoma model following treatment with NBMs

**Fig. 13:**
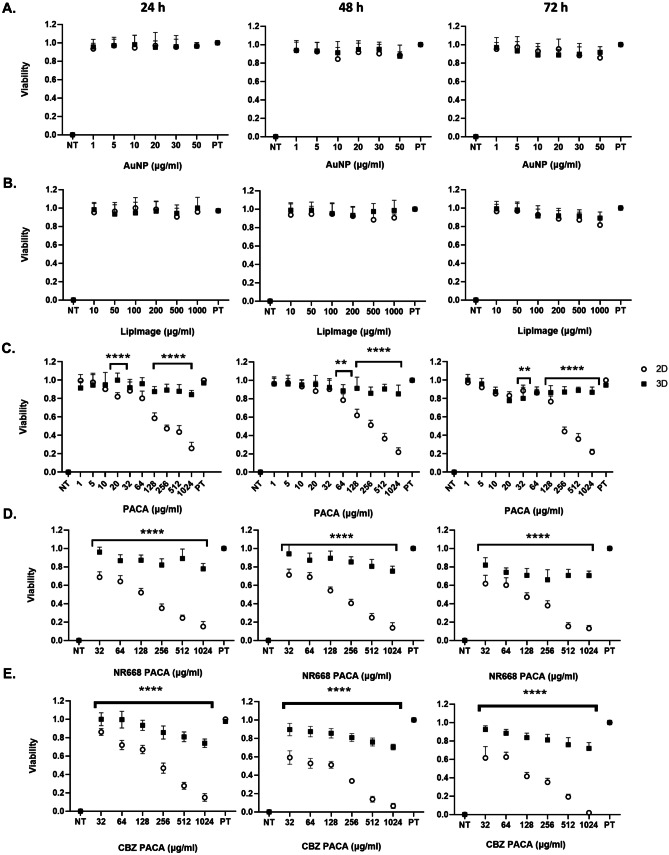
Evaluation of cell viability in NBM-treated hepatic 2D monocultures and 3D hepatic spheroids at 3 different exposure timepoints using the CellTiter-Glo^®^ ATP quantification assay. **A** AuNP, **B** LipImage^™^ 815, **C** poly(alkyl cyanoacrylate) (PACA), **D** NR668-loaded poly(alkyl cyanoacrylate) (PACA) and **E** CBZ-loaded poly(alkyl cyanoacrylate) (PACA) all assessed after 24-, 48- and 72-h exposure. Data is expressed as fraction of viable cells; data normalised to 1.0 for positive control (PT–untreated cells) and representative of a minimum of three independent experiments (*n* = 3) and expressed as mean ± SD. *p < 0.05, **p < 0.01, ***p < 0.001 and ****p < 0.0001. Statistical analysis by two-way ANOVA, with post hoc Sidak test. All graphs and statistical analysis were undertaken using GraphPad Prism 9, Version 9.4.0 (GraphPad Software, Inc, San Diego, USA)

The effects of AuNP, LipImage^™^ 815 and three poly(alkyl cyanoacrylate) (PACA) NBM formulations (i.e. unloaded, dye-loaded and drug (CBZ) loaded) on the viability of HepG2 cells in 2D and 3D cultures were measured after 24-h, 48-h and 72-h exposure, using the CellTiter-Glo^®^ luminescent viability assay (Fig. 13). There is no significant reduction in viability in 2D and 3D cultures from AuNP and LipImage^™^ 815 (Fig. 13A and B). Significant differences in cell viability were observed however between both culture types following treatment with all poly(alkyl cyanoacrylate) (PACA) formulations (unloaded, dye-loaded and drug (CBZ) loaded) (Fig. 13C, D and E), most notably as concentrations increased. The largest reduction in viability in 2D culture was seen with CBZ-loaded PACA.

### Cytotoxicity induced by NBMs in 2D and 3D hepatocellular carcinoma models

**Fig. 14:**
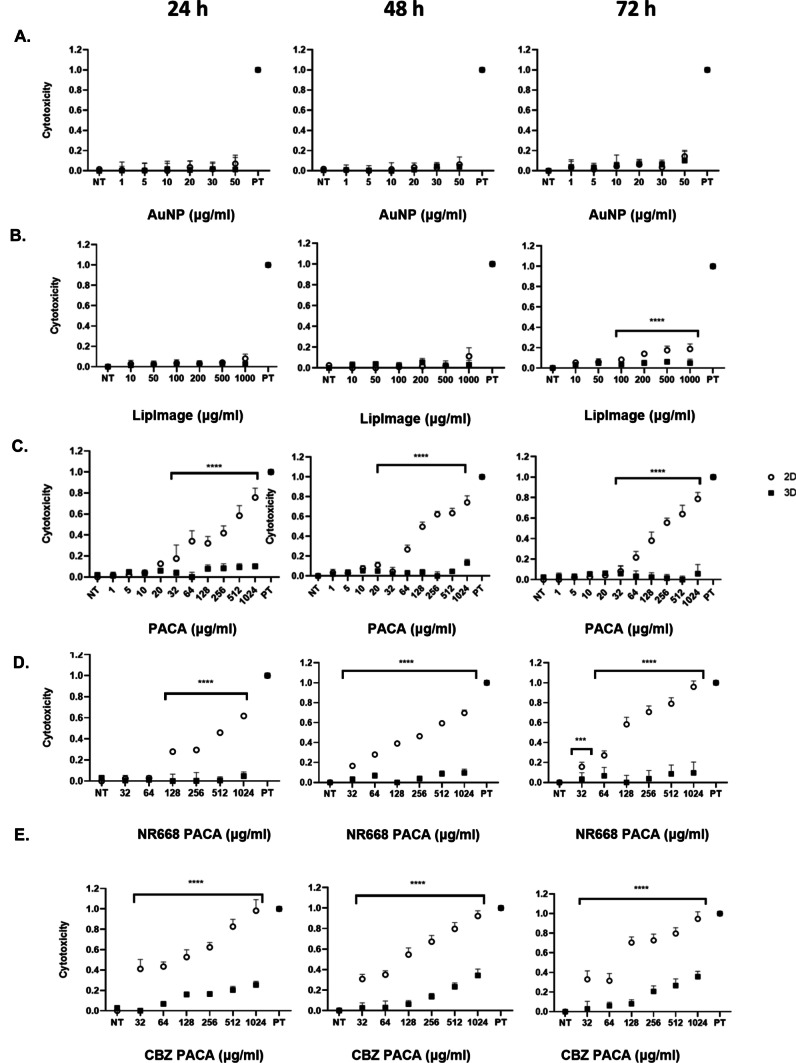
Evaluation of cytotoxicity induced by NBMs in hepatic 2D monocultures and 3D hepatic spheroids at 3 different exposure timepoints using the LDH cytotoxicity assay. **A** AuNP, **B** LipImage^™^ 815, **C** PACA, **D** NR668-loaded PACA and **E** CBZ-loaded PACA, all assessed after 24-, 48- and 72-h exposure. Data is expressed as fraction of cells lysed using lysis buffer (1.0 = 100%; PT) and representative of a minimum of three independent experiments (*n* = 3) and expressed as mean ± SD. *p < 0.05, **p < 0.01, ***p < 0.001 and ****p < 0.0001. Statistical analysis by two-way ANOVA, with post hoc Sidak test. All graphs and statistical analysis were undertaken using GraphPad Prism 9, Version 9.4.0 (GraphPad Software, Inc, San Diego, USA)

Following the assessment of cell viability, the toxic effects of each NBM were quantitatively assessed using the LDH assay. Here, the amount of LDH enzyme that leaks out from the plasma membrane of damaged cells is detected. As determined by both 2D and 3D culture supernatants, no significant cytotoxicity was observed in 2D and 3D cultures following exposure to both AuNP and LipImage^™^ 815 (Fig. 14A and B). Significant dose-dependent cytotoxicity was observed in all PACA samples, most specifically for CBZ-PACA cultures and in particular at larger concentrations (Fig. 14C, D and E).

**Fig. 15:**
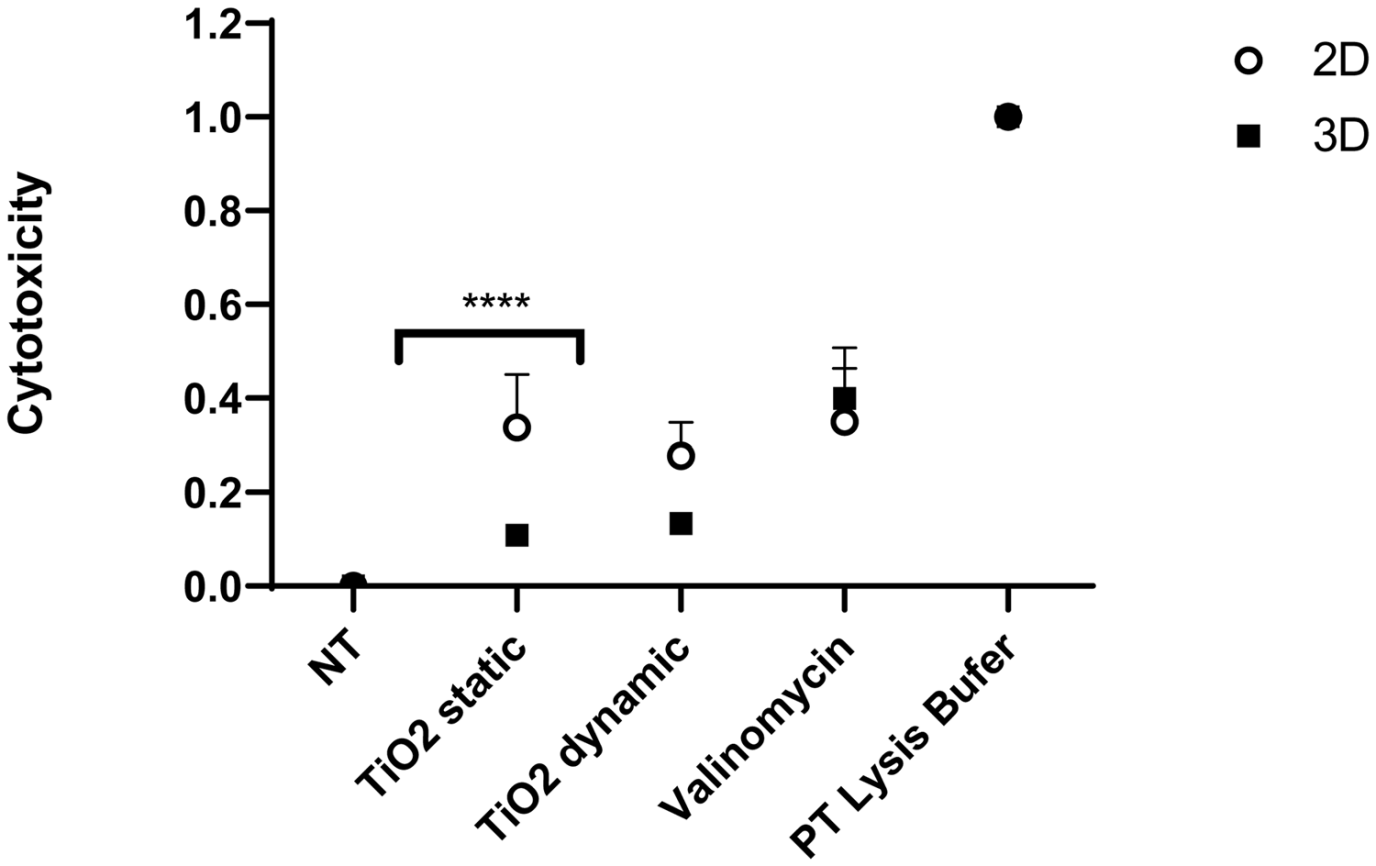
Experimental cytotoxicity assessment between static and dynamic conditions by means of comparison between control samples after 24 h using the LDH assay. NT represents no treatment (cell culture medium only), TiO_2_ represents 10 µg/ml nanoparticle positive control tested in both static and dynamic conditions using a Mimetas rocker, valinomycin represents a 125 µM valinomycin experimental positive control and PT represents 50 µl 10 × lysis buffer assay positive control provided with LDH kit. Data is expressed as fraction of lysed cells (1.0 = 100%; PT) and representative of a minimum of three independent experiments (*n* = 3) and expressed as mean ± SD. *p < 0.05, **p < 0.01, ***p < 0.001 and ****p < 0.0001. Statistical analysis by two-way ANOVA, with post hoc Sidak test. All graphs and statistical analysis were undertaken using GraphPad Prism 9, Version 9.4.0 (GraphPad Software, Inc, San Diego, USA)

Furthermore, the relationship between static vs. dynamic regimen experimental condition was also evaluated by measuring the expected responses of the chosen positive and negative controls, as shown in Fig. 15.

## Discussion

In this study, a 3D hepatic cell culture model was introduced, characterised and then used for the testing of a variety of NBM formulations. Whilst the current gold standard for drug metabolism and toxicity studies is the use of primary human hepatocytes (PHH) [32, 33], their short life span, high costs, limited availability and the inter-donor variation observed significantly limit their use in high-throughput in vitro toxicity screening. For this reason, the immortalised hepatocyte cell line HepG2 was used for this work. Although, when cultured in 2D, HepG2 cells exhibit many limitations including loss of liver-specific structure and functionality [32, 34], their ease-of-use as compared to PHH, coupled with a stable phenotype and no donor variation [32, 35], offers many advantages for the development of standard operating procedures (SOPs) for pre-clinical screening applications. Furthermore, it has been shown that cultured in 3D environments, HepG2 cells regain some of the characteristics they lose when cultured in 2D. In 3D, HepG2 cells exhibit reduced proliferation rates, self-organise and differentiate to form 3D spheroids, which regain lost hepatic structure and function [36, 37].

For this study, HepG2 spheroids were cultured in CellStar^®^ cell-repellent plates and characterised, before being used in the assessment of five chosen NBMs. These plates were chosen as they are easy to acquire, standardised and high throughput in nature, whilst also known to produce reproducible spheroids [38–40]. HepG2 spheroids formed successfully using this method, with the cells in each well aggregating to form a single spheroid. Spheroids formed from this method could be rapidly cultured by directly seeding into the plate, without the need for complicated transferring of spheroids or expensive hanging drop plates, or the need for much optimisation, and they were cost-effective and demonstrated a higher degree of reproducibility in forming 3D HepG2 spheroids. As presented in Fig. 8 (Brightfield) and Fig. 9 (H&E), spheroids formed from a cell seeding concentration of 1,000 cells per 100-µl culture medium and cultured for 7 days, the chosen timepoint for further experimentation, had defined perimeters, limited cell death and compact structure. The production and secretion of various materials including albumin, ammonia and urea in hepatocytes are key characteristics of their functionality and high indicative of long-term performance [41]. From undertaking a human serum albumin ELISA, it could be confirmed that HepG2 spheroids were in fact synthesising and secreting albumin, in significantly higher quantities than 2D cultures (Fig. 11). Increased albumin secretion was also observed for the largest cell density, i.e. 10000. It can be hypothesised that this is due to the increased cell number. The synthesis of albumin in HepG2 spheroids indicates synthetic and metabolic function as production of albumin is vitally important for hepatocyte physiological function in vivo [42].

One of the major uses of hepatic spheroids in pre-clinical research and one of their most exciting and useful applications today is their ability to predict hepatotoxicity of various drugs and materials. It has been suggested that many compounds induce liver injury through damaging the mitochondria, including fialuridine, diclofenac, troglitazone and amiodarone [43–47]. Other hepatotoxins can cause transporter dysfunction or loss of hepatocyte structural integrity, such as tolcapone, nefazodone, troglitazone and diclofenac [43, 44, 46]. Because of this, the secretion of bile is hindered and does not effectively get secreted into bile ducts. This causes toxic substances to build-up around hepatocytes [45, 46, 48–50]. Outside of mitochondria and bile duct dysfunction, metabolites of certain compounds often cause damage to the liver, either by being directly hepatotoxic or by inducing the formation of adducts with proteins in the liver. Common compounds which induce toxicity in this manner include tolcapone, troglitazone, nefazodone, acetaminophen and diclofenac [51, 52]. Other studies have suggested that immune response can be involved, elevating the toxicity of a variety of hepatotoxins, including diclofenac and trovafloxacin [53–55]. However, for any of the aforementioned damages to be detected appropriately, sufficient cell communication and signalling must be present in the model, along with the ability to initiate immune responses. To probe whether HepG2 spheroids would be better predictors of hepatotoxicity than 2D cultures, spheroids and HepG2 monolayers were treated with varying concentrations of four common hepatotoxic compounds, namely acetaminophen, diclofenac, fialuridine and trovafloxacin. Following a protocol detailed in Gaskell et al., a 4-day repeat-dosing regimen was used to expose 3D spheroids. For 2D monolayers, however, acute dosing only was assessed as the monolayers became over-confluent and exhibited reduced viability after 48 h in culture. After treatment with the hepatotoxic compounds, a dose-dependent reduction in ATP production was observed in spheroids (Fig. 12). Each of the five chosen compounds was found more toxic in 3D spheroids compared to the 2D monocultures, although differences were not statistically significant. The concentration causing a 50% reduction in cell viability (IC_50_) was estimated from the graphs for each drug tested and is reported in Table 7, with comparison made to data extracted from the scientific literature also reported. Each of the four hepatotoxic compounds screened in this study exhibited more toxicity and greater sensitivity in HepG2 spheroids when compared to the 2D monolayer. Also, it was found that spheroid culture time had no effect on these results, shown for spheroids cultured for both 5 and 12 days (Supplementary Fig. 1). Greater sensitivity is also reported between 2D HepG2 monolayers, and values quoted in literature for HepG2 2D monolayers by both Bort et al. (diclofenac) [56] and Ju et al. (acetaminophen) [57]; however, some differences in the methodologies used may account for these differences, for instance, the use of a microfluidics devise by Ju et al. Interestingly, it can be hypothesised that this increased toxicity in spheroids is due to the direct cell–cell contacts, increased liver-specific functionality and structure of the HepG2 spheroids allowing the hepatotoxins to exert their effects, in comparison to the 2D monolayers, with their limited monolayer planar cell contacts and lack of transporter functionality. Various other studies have also found that 3D hepatic spheroids are greater predictors of hepatotoxicity than 2D monolayers, supporting the results in this study and the hypothesis that culturing hepatic cells as 3D spheroids increases hepatotoxin sensitivity [58–60]. For example, Li et al. used 3D hepatic spheroids comprised of PHH to assess the hepatotoxic potential of 100 known hepatotoxic drugs, and found that in comparison to conventional 2D liver monolayers, the 3D spheroids were significantly more sensitive to detecting hepatotoxicity [61]. Bell et al. have detailed similar findings [62], as have Ramaiahgari et al., who used HepG2 spheroids [63]. Whilst HepG2 spheroids were more predictive than HepG2 2D monolayers to all hepatotoxins, from values based in literature and from my own experiments, estimated values at which 50% cell viability is reduced were only comparable to PHH for trovafloxacin and diclofenac treatments, and not for acetaminophen or fialuridine. It may be hypothesised that the mechanisms for which both acetaminophen and fialuridine exhibit their hepatotoxicity play a role in this variation. With regard to acetaminophen, it is suggested that metabolites of this compound cause liver damage, either by being directly hepatotoxic or by forming adducts with various liver proteins. As CYP enzyme activity and the ability to metabolise xenobiotic compounds were not probed in this study, it can be hypothesised that the variation observed between HepG2 spheroids and PHH may potentially stem from the lack of drug metabolites forming in HepG2 spheroids and the ability of the spheroids to observe their toxic effects [64]. This will be probed in future work. It has also been suggested that various other compounds induce hepatotoxicity through mitochondrial damage, including amiodarone and troglitazone, and the hepatotoxin used in this study, fialuridine [45, 65, 66]. Here, variation is observed between HepG2 spheroids and PHH when treated with fialuridine. The presence of functional mitochondria in HepG2 spheroids was also not probed in this study, and the inclusion of mitochondria assessment in further work would be of great benefit to underpin the toxicity of fialuridine in the spheroid model presented. It is also suggested that immune response is involved in hepatotoxicity, going so far as to accentuate hepatotoxicity of certain compounds. Both diclofenac and trovafloxacin, the two hepatotoxins used in this study which exhibited similar sensitivity to PHH, are compounds which display evidence of inflammatory or immune-mediated toxicity [47, 51]. For this hepatotoxicity to be successfully detected in spheroids, proper cell communications and signalling must be present in the model, as well as capabilities to either alert or initiate immune responses. The comparable sensitivity of the HepG2 spheroids and PHH suggests that this is indeed the case in the HepG2 spheroids, and that cell communication and signalling may be comparable to in vivo*.*

Finally, the impact five NBMs had on cell viability and any induction of cytotoxicity was assessed up to 72-h exposure. To overcome one of the key issues seen with the pre-clinical assessment of NBMs, being interference with assays and/or reagents, in this study, the CellTiter-Glo^®^ ATP quantification, a frequently used assay for 3D microtissues, was used for determining both 2D and 3D spheroid viabilities. Here, treated spheroids are incubated with CellTiter-Glo^®^ reagent and viability is judged by the quantification of a luminescent signal formed when luciferin is converted by luciferase, which is in turn indicative of cytoplasmic ATP concentration. This reagent successfully penetrates even large spheroids and has an increased lytic capacity, allowing the time effective, standardised and more accurate determination of viability in 3D structures when compared to other standard methods [67]. In parallel to assessing viability using ATP quantification, the LDH assay was used to determine NBM-induced cytotoxicity. As the LDH assay is supernatant based, with the cytosolic enzyme LDH released into culture supernatants by compromised plasma membranes, this assay avoids issues with having to dissociate the spheroid structure as is necessary for other membrane integrity assessment methods like neutral red or trypan blue. When assessed using these assays, no reduction of viability (Fig. 13) or induction of cytotoxicity (Fig. 14) was observed following treatments with both AuNP and LipImage^™^ 815 in both culture types. This is in agreement with various studies who have shown that similar sized/coated AuNPs and LipImage^™^ 815 are generally biocompatible and well tolerated [68]. In comparison to this, each PACA NBM induced a dose-dependent reduction in viability and increase in cytotoxicity at all timepoints, most notable after 72 h, in 2D cultures. A similar toxicity profile was observed in a previous published work from Sulheim et al. [27]*.* The same pattern was not observed in 3D cultures, with significant differences found between both culture types. It can be said that this result is observed and recorded, due to the more in vivo-like phenotype of the 3D spheroids and not due to the material type, as determined by TiO_2_ control (Fig. 15). The most toxic NBM was found to be the CBZ-loaded PACA, with an increase in cytotoxicity and a reduction in viability also observed in 3D cultures for this NBM.

As traditional 2D cell culture models do not adequately represent the structure or the function of 3D hepatic tissue, which has extensive and complex cell–cell and cell–matrix interactions, and shows vastly different diffusion and transport conditions, viability and cytotoxicity assessments of NBM-treated liver cultures in two dimensions do not accurately reflect the actual toxicity of NBMs in the body. Therefore, the development of advanced 3D hepatocellular carcinoma models for the toxicity screening of these materials in vitro is vitally important in order to have more in vivo-like, realistic models that have tissue like physiology, for their risk assessment. Whilst hepatic spheroids have become more popular in recent decades for assessing issues like drug-induced liver injury (DILI), their uses in the study and pre-clinical assessment of NBMs are still in its infancy. In the past couple of years, and in particular in 2020, various publications have acknowledged the potential they hold for accurately assessing nanomaterials in the pre-clinical in vitro evaluation stage. Publications from Dubiak-Szepietowsk et al. [69], Elje et al. [70, 71] and Fleddermann et al. [72] have all utilised hepatic spheroids for assessing distribution or toxicity of NPs. The materials in these studies are however encountered as bulk materials, assessed for occupational or environmental safety, and thus they are not NBMs developed for medical uses or medical technology, like the materials presented in this study. To date, very few publications have used 3D hepatocellular carcinoma models for assessing toxicity of NBMs, with most studies utilising spheroids to assess depth and penetrations of NBMs in 3D [73–77]. To the best of our knowledge, this study is the first time 3D liver spheroids have been used in the in vitro toxicity screening of these specific NBMs, making the work presented an advancement in the nanomedicine field. In this study, a 3D hepatic cell culture model was introduced and its applicability to the pre-clinical assessment of NBMs tested. Whilst two materials exhibited no significant differences between the two culture types, due to their overall biocompatibility, great differences were seen between 2 and 3D following treatment with the PACA NBMs, illustrating the importance of testing NBMs in more than one culture setting. With all this being said, the HepG2 3D spheroids presented in this work can be described as a promising and human-relevant model, amenable for the accurate and sensitive screening of NBMs in vitro, whilst also being compliant with the 3Rs policy to reduce in vivo animal testing. As HepG2 spheroids remain viable for long periods, they are also amenable for repeated or chronic dosing regimens, with the possibility to analyse both sub-acute and delayed toxic effects. Overall, the work presented here provides a positive contribution to the development of advanced in vitro models for screening the safety of NBMs.

## Supplementary Information

Below is the link to the electronic supplementary material.

Supplementary file1 (DOCX 51 KB)

## Data Availability

The data that support the findings of this study are available from the corresponding author (MAT), on special request.
