# Supplementary material for: Pre-clinical 2D and 3D toxicity response to a panel of nanomaterials; comparative assessment of NBM-induced liver toxicity
Source: Drug Deliv Transl Res. 2022 Jun 28;12(9):2157–77. doi: 10.1007/s13346-022-01170-1 (PMC9360078; doi:10.1007/s13346-022-01170-1)
Supplement: Supplementary file 1 — Supplementary file1 (DOCX 51 KB) [file 13346_2022_1170_MOESM1_ESM.docx]

# Pre-clinical 2D and 3D toxicity response to a panel of nanomaterials; Comparative assessment of NBM-induced liver toxicity

Melissa Anne Tuttya,b, Gabriele Vellaa,b,, Adriele Prina-Melloa,b,c

*a Nanomedicine and Molecular Imaging Group, Trinity Translational Medicine Institute (TTMI), School of Medicine, Trinity College Dublin, Dublin 8, Ireland*

*b Laboratory for Biological Characterisation of Advance Materials (LBCAM), TTMI, School of Medicine, Trinity College Dublin, Dublin 8, Ireland*

*c Trinity St James’s Cancer Institute, Trinity College Dublin, St James’s Hospital, Dublin 8, Ireland*

**SUPPLEMENTARY INFORMATION**

Corresponding Authors:

*Dr. Melissa Anne Tutty

Email: [tuttym@tcd.ie](mailto:tuttym@tcd.ie)

ORCID: 0000-0002-6705-2492

*Dr. Adriele Prina-Mello

Email: [prinamea@tcd.ie](mailto:prinamea@tcd.ie)

ORCID: 0000-0002-4371-2214

**Keywords:** nanobiomaterials; 3D culture; spheroids; cytotoxicity; viability; HepG2; liver spheroid; 3Rs.

**Supplementary Figure 1**


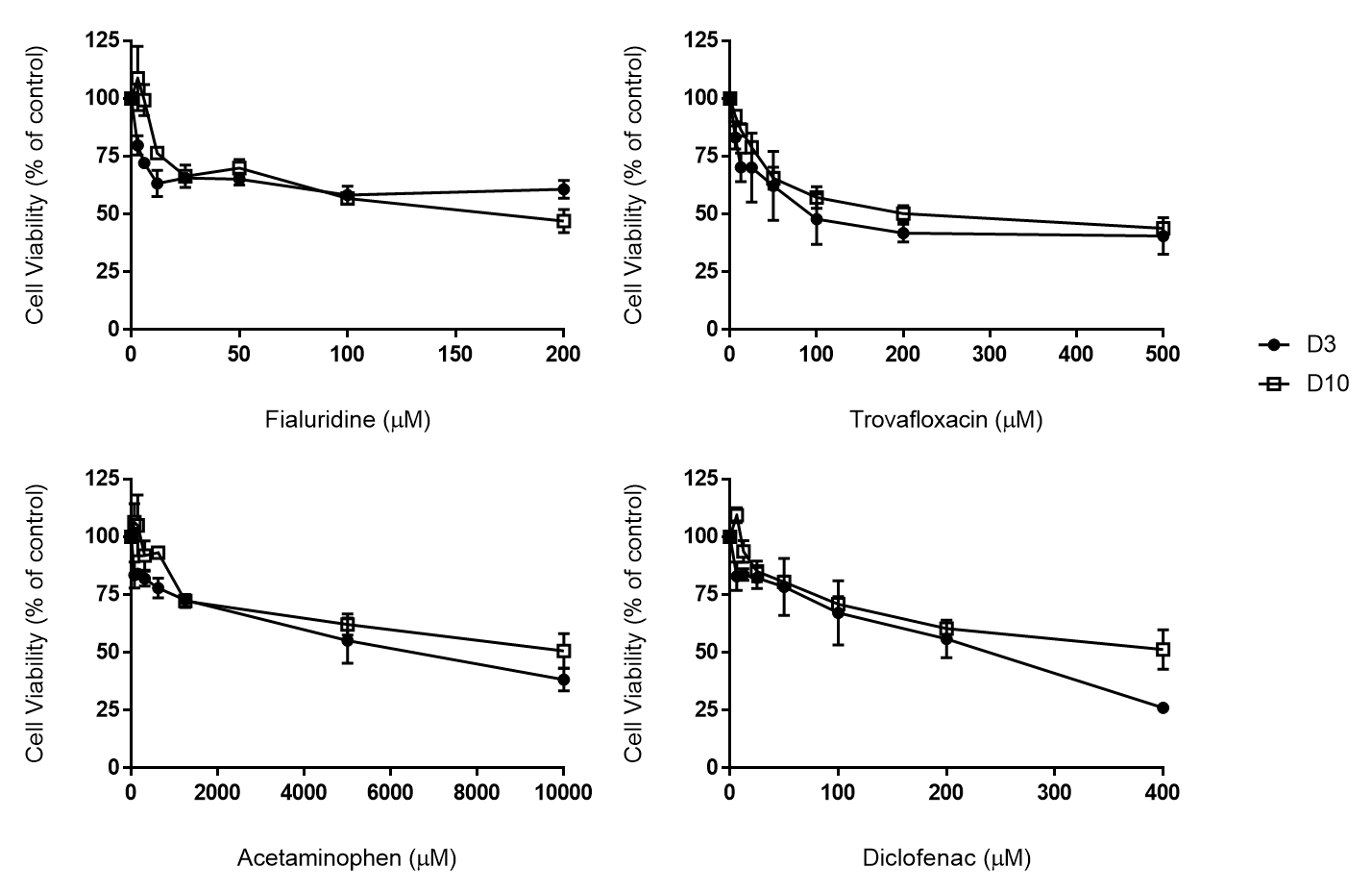


**Supplementary Figure 1. Effect of culture time on spheroid response to hepatotoxins.** Spheroids were cultured for both 3-day or 10-day and then incubated with the four chosen hepatotoxins (acetaminophen, diclofenac, fialuridine, and trovafloxacin) with repeated dosing at day 5 and day 12. Cell viability was determined using ATP quantification and results plotted as a percentage of untreated control. Data are represented as mean ± SEM (n=3 in duplicate). Changes were not statistically different (multiple t-tests with Bonferroni Dunn method).
